# Environmental pH stress influences cellular secretion and uptake of extracellular vesicles

**DOI:** 10.1002/2211-5463.13107

**Published:** 2021-02-18

**Authors:** Ikuhiko Nakase, Natsumi Ueno, Mie Matsuzawa, Kosuke Noguchi, Mami Hirano, Mika Omura, Tomoya Takenaka, Ayaka Sugiyama, Nahoko Bailey Kobayashi, Takuya Hashimoto, Tomoka Takatani‐Nakase, Eiji Yuba, Ikuo Fujii, Shiroh Futaki, Tetsuhiko Yoshida

**Affiliations:** ^1^ Graduate School of Science Osaka Prefecture University Sakai Japan; ^2^ NanoSquare Research Institute Osaka Prefecture University Sakai Japan; ^3^ Keio University School of Medicine Tsukuba Japan; ^4^ Institute for Advanced Sciences Toagosei Co., Ltd Tsukuba Japan; ^5^ Department of Applied Chemistry Graduate School of Engineering Osaka Prefecture University Sakai‐shi Japan; ^6^ Department of Pharmaceutics School of Pharmacy and Pharmaceutical Sciences Mukogawa Women's University Nishinomiya Japan; ^7^ Institute for Bioscience Mukogawa Women's University Nishinomiya Japan; ^8^ Institute for Chemical Research Kyoto University Uji Japan

**Keywords:** cell‐penetrating peptides, cellular uptake, exosomes, extracellular vesicles, pH

## Abstract

Exosomes (extracellular vesicles/EVs) participate in cell–cell communication and contain bioactive molecules, such as microRNAs. However, the detailed characteristics of secreted EVs produced by cells grown under low pH conditions are still unknown. Here, we report that low pH in the cell culture medium significantly affected the secretion of EVs with increased protein content and zeta potential. The intracellular expression level and location of stably expressed GFP‐fused CD63 (an EV tetraspanin) in HeLa cells were also significantly affected by environmental pH. In addition, increased cellular uptake of EVs was observed. Moreover, the uptake rate was influenced by the presence of serum in the cell culture medium. Our findings contribute to our understanding of the effect of environmental conditions on EV‐based cell–cell communication.

## Graphical abstract

Extracellular vesicles (EVs) participate in cell–cell communication. Here, we report that pH stress significantly affected the secretion of EVs with increased protein content and zeta potential. In addition, increased cellular uptake of EVs, which were secreted under conditions of pH stress, was observed. Our findings contribute to our understanding of the effect of environmental conditions on EV‐based cell–cell communication.

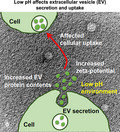

**Abbreviations**
ALIX: ALG‐2‐interacting protein X
DMF: *N*,*N*‐dimethylformamide
EMCS: *N*‐ε‐maleimidocaproyl‐oxysulfosuccinimide ester
ESCRT: endosomal sorting complexes required for the transport
EVs: extracellular vesicles
FITC: fluorescein‐5‐isothiocyanate
Fmoc: 9‐fluorenylmethyloxycarbonyl
GABA: γ‐aminobutyric acid
MALDI‐TOFMS: matrix‐assisted laser desorption/ionization time‐of‐flight mass spectrometry
MVBs: multivesicular bodies
NMM: *N*‐methylmorpholine
TEM: transmission electron microscope
TIRF: total internal reflection fluorescence

Exosomes (extracellular vesicles, EVs), which are secreted from various types of cells such as normal and disease‐related cells, play crucial roles in cell–cell communication [1, 2]. EVs contain from cellular membranes generated in multivesicular bodies (MVBs) and are 30–200 nm in diameter [1, 2]. EVs consist of lipids (sphingomyelin, cholesterol, ceramide), membrane proteins (Alix, TSG 101), tetraspanins (CD63, CD37, CD53, CD81, CD82), heat‐shock proteins (Hsp84/90, Hsc70), antigens (MHC I and MHC II), enzymes (phosphate isomerase, peroxiredoxin, aldehyde reductase, fatty acid synthase), and microRNAs [1, 3]. In previous immunotherapy trials, the potential of EV‐based cancer vaccines (e.g., antigens on EVs recognize and destroy cancer cells) has been explored [4, 5, 6, 7, 8]. Bioactive molecules such as microRNAs and enzymes are capsulated inside the secreted EVs, which are internalized by surrounding cells, affecting cellular functions and aiding disease progression [1, 2, 3, 9, 10, 11]. In addition, cancer‐type‐specific microRNAs encapsulated in the secreted EVs have been found as, for example, miR‐21 and miR‐141 in ovarian cancer, miR‐107 and miR‐130b in prostate cancer, and next‐generation cancer diagnostic technologies for detecting cancer by analyzing EV contents are highly expected with minimal burden on patients, due to the ease of collection of EVs from the patient's bodily fluids, such as blood and urine [1, 2, 3, 9, 10, 11].

Endocytosis has been proposed to be the predominant mechanism for the cellular uptake of EVs, and EV membrane proteins (e.g., CD9, CD81) have been reported to act as ligands for EV endocytosis [12, 13, 14, 15]. Recently, our research group reported that macropinocytosis induced by the activation of epidermal growth factor receptor or the expression of oncogenic K‐Ras is a crucial pathway for the efficient cellular uptake of EVs [16]. Because of their pharmaceutical advantages, EVs are also being utilized as intracellular delivery carriers of therapeutic molecules. The proposed pharmaceutical applications take advantage of EVs to achieve, for example, encapsulation of natural and/or artificial therapeutic/diagnostic molecules inside EVs, controlled immunoreaction, effective usage of cell–cell communication routes of EVs, blood‐brain barrier penetration, infinite secretion, and expression of functional proteins [17, 18, 19, 20, 21, 22].

Extracellular vesicles are generated in MVBs in cells as intraluminal vesicles and are formed through inward budding of the MVB membranes [2, 23]. Cytoplasmic molecules are selectively loaded into the MVBs [2]. In the exocytic pathway, the MVBs are fused with the plasma membrane prior to the release of EVs to the extracellular space. Rab GTPases such as Rab11/35, Rab7, and Rab27 have been shown to be involved in EV secretion [2]. Endosomal sorting complexes required for the transport (ESCRT)‐dependent pathway (multiprotein complexes of ESCRT‐0, ESCRT‐I, ESCRT‐II, and ESCRT‐III) have been shown to play crucial roles in the mechanism of EV biogenesis [24, 25, 26]. Complexes of ESCRT‐0, ESCRT‐I and ESCRT‐III recognize ubiquitinated cargo. Next, the ESCRT‐III complex transiently assembles on endosomes and conducts vesicle scission [25]. Recently, auxiliary components such as ATPase, vacuolar protein sorting‐associated protein, and ALG‐2‐interacting protein X (ALIX) have been shown to participate in the ESCRT membrane‐scission machinery [25]. In addition, the budding and/or release of EVs has been shown to be regulated by lipids, including sphingolipid ceramide [24] and sphingosine 1 phosphate, in an ESCRT‐independent pathway [26]. Furthermore, Parolini and collaborators reported that pH reduction in the cell environment increased the efficacy of EV secretion [27]. In addition, the cellular uptake of the EVs through membrane fusion increased in melanoma cells grown in low pH conditions [27]. However, detailed characteristics of secreted EVs produced by cells grown in low pH conditions are still unknown.

In this study, we found that the low pH significantly increased protein content of secreted EVs having increased zeta potential of EV membranes, even though pH reduction around the cells decreased the cellular proliferation rate. Interestingly, the expression level and location of GFP‐fused CD63, which is one of the marker proteins (tetraspanin) present on EVs (exosomes), were also significantly affected in cells by the environmental pH, and enhanced cellular uptake of EVs, which were isolated from cells cultured in a low pH medium, was also observed (Fig. 1). In addition, the modification of arginine‐rich cell‐penetrating peptides, which have been shown to effectively induce macropinocytotic cellular uptake, on the isolated EVs resulted in further enhancement in cellular uptake of EVs, suggesting essential factors of membrane components for deciding cellular uptake efficacy of EVs and demonstrating a useful technique for intracellular delivery using the EVs.

**Fig. 1:**
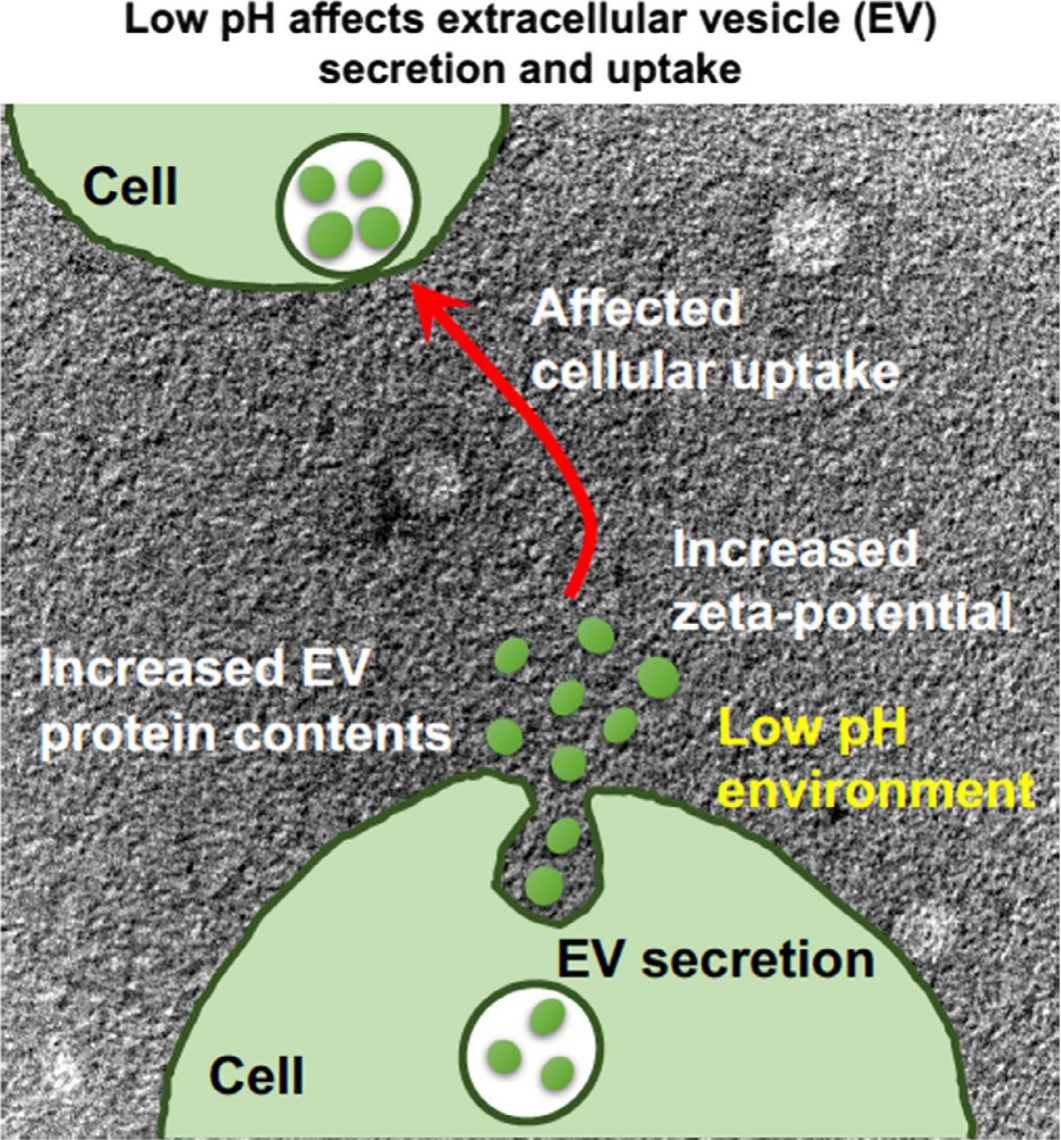
Schematic of our research findings: low pH affects EV secretion and cellular uptake.

## Materials and methods

### Peptide synthesis

Chemical synthesis of all peptides was conducted via 9‐fluorenylmethyloxycarbonyl (Fmoc) solid‐phase peptide synthesis on a Rink amide resin with the coupling reagents 1‐hydroxybenzotriazole/2‐(1H‐benzotriazole‐1‐yl)‐1,1,3,3‐tetramethyluronium hexafluorophosphate (Peptide Institute, Osaka, Japan)/*N*‐methylmorpholine (NMM) as previously described [28, 29]. The Rink amide resin and the Fmoc amino acid derivatives were purchased from Shimadzu Biotech (Kyoto, Japan) and the Peptide Institute, respectively. To prepare the acetylated peptide, the N terminus of the peptide resin was acetylated using acetic anhydride in the presence of NMM in dimethylformamide (DMF), as previously reported [30]. Deprotection of the protected peptide and cleavage from the resin was conducted via treatment with a trifluoroacetic acid/ethanedithiol mixture (95 : 5) for 3 h at 25 °C, followed by reverse‐phase HPLC purification. The purity of each peptide was estimated to be > 97% on the basis of the analytical HPLC. The structures of the synthesized peptides were confirmed using matrix‐assisted laser desorption/ionization time‐of‐flight mass spectrometry (MALDI‐TOFMS; Microflex, Bruker, Billerica, MA, USA).

#### Ac‐CG‐R8 (CH_3_‐CO‐NH‐Cys‐Gly‐(Arg)_8_‐amide)

MALDI‐TOFMS: 1469.0 [calcd. for (M + H)^+^: 1468.9]. Retention time in HPLC was 9.6 min [column: Cosmosil 5C18‐AR‐II (4.6 × 150 mm); gradient: 5–50% B in A (A = H_2_O containing 0.1% CF_3_COOH, B = CH_3_CN containing 0.1% CF_3_COOH) over 30 min; flow: 1 mL·min^−1^; detection: 220 nm]. Yield from the starting resin was 19.8%.

Conjugation of peptides was with *N*‐ε‐maleimidocaproyl‐oxysulfosuccinimide ester (EMCS) linker. For the preparation of EMCS linker‐conjugated peptides, each purified Ac‐CG‐R8 peptide was reacted with EMCS (1.1 equivalents; Thermo Fisher Scientific Inc., Rockford, IL, USA) in DMF for 2 h at room temperature followed by HPLC purification.

#### EMCS‐R8 (CH_3_‐CO‐NH‐Cys(EMCS)‐Gly‐(Arg)_8_‐amide)

MALDI‐TOFMS: 1856.7 [calcd. for (M + H)^+^: 1856.2]. Retention time in HPLC was 9.5 min [column: Cosmosil 5C18‐AR‐II (4.6 × 150 mm); gradient: 5–95% B in A (A = H_2_O containing 0.1% CF_3_COOH, B = CH_3_CN containing 0.1% CF_3_COOH) over 30 min; flow: 1 mL·min^−1^; detection: 220 nm]. Yield from the starting resin was 12.5%.

For the preparation of fluorescently labeled peptides, a peptide resin with γ‐aminobutyric acid (GABA) at its N terminus was prepared, and the N terminus was modified with fluorescein‐5‐isothiocyanate (FITC) in the presence of *N*,*N*‐diisopropylethylamine in DMF [31]. Deprotection of the protected peptide, cleavage from the resin, and HPLC purification were conducted as mentioned above.

#### FITC‐EMCS‐R8 (FITC‐NH‐GABA‐Cys(EMCS)‐Gly‐(Arg)_8_‐amide)

MALDI‐TOFMS: 2289.4 [calcd. for (M + H)^+^: 2288.7]. Retention time in HPLC was 12.4 min [column: Cosmosil 5C18‐AR‐II (4.6 × 150 mm); gradient: 5–95% B in A (A = H_2_O containing 0.1% CF_3_COOH, B = CH_3_CN containing 0.1% CF_3_COOH] over 30 min; flow: 1 mL·min^−1^; detection: 220 nm). Yield from the starting resin was 2.8%.

### Cell cultures

The human epidermoid carcinoma‐derived A431 cells were purchased from the American Type Culture Collection (Manassas, VA, USA). The cells were cultured in minimum essential medium (MEM; Gibco, Life Technologies Corporation, Grand Island, NY, USA) containing 10% heat‐inactivated FBS (Gibco, Life Technologies Corporation). HeLa (human cervical cancer‐derived) cells were purchased from the Riken BRC Cell Bank (Ibaraki, Japan). The cells were cultured in α‐MEM (Gibco, Life Technologies Corporation) containing 10% heat‐inactivated FBS (Gibco, Life Technologies Corporation). The cells were grown on 100‐mm dishes and incubated at 37 °C under 5% CO_2_.

### Preparation of HeLa cells stably expressing GFP‐fused CD63

CD63 is a membrane marker tetraspanin protein of exosomes, and we prepared HeLa cells stably expressing the GFP‐fused CD63 to secrete CD63‐GFP‐containing EVs (CD63‐GFP‐EVs) as previously reported [16]. HeLa cells (1 × 10^5^ cells) were plated on a 24‐well microplate (Iwaki, Tokyo, Japan) and incubated for 1 day. They were transfected with CD63‐GFP plasmid (pCT‐CD63‐GFP, pCMV, Cyto‐Tracer; System Biosciences, Mountain View, CA, USA; 800 ng) complexed with Lipofectamine LTX reagent (2 μL) and PLUS reagent (1 μL; Invitrogen, Life Technologies Corporation) in α‐MEM containing 10% FBS (200 μL). The cells were also treated with puromycin (3 μg·mL^−1^; LKT Laboratories, St. Paul, MN, USA) for the antibiotic selection of HeLa cells stably expressing CD63‐GFP (CD63‐GFP‐HeLa).

### Isolation of EVs

CD63‐GFP‐HeLa cells (2 × 10^6^ cells) were plated onto 100‐mm dishes in 10% FBS containing α‐MEM for 1 day at 37 °C under 5% CO_2_. The cells were washed with α‐MEM without FBS (3 mL, three times) and were incubated with 10% EV‐free FBS (EXO‐FBS, ATLAS biological, Fort Collins, CO, USA) containing α‐MEM (pH 5, 6, or 7; 10 mL per dish) for 48 h at 37 °C under 5% CO_2_. Secreted EVs in the cell culture medium were isolated using ultracentrifugation as previously reported [21, 22]. The collected cell culture medium was centrifuged (300 ***g***) for 10 min at 4 °C. The supernatant was centrifuged (2000 ***g***) for 10 min at 4 °C and again centrifuged (10 000 ***g***) for 30 min at 4 °C to remove cell debris. The supernatant was then centrifuged (100 000 ***g***) for 2 h at 4 °C (Himac CP85β; Hitachi, Tokyo, Japan) in duplicate, and the pellet was collected in PBS. The concentrations of isolated EVs are shown in terms of their protein concentrations, which were determined using a Pierce BCA protein assay kit (Thermo Fisher Scientific Inc.). The number of EV particles was detected using NanoSight (NanoSight Ltd., Minton Park, Amesbury, UK).

### Western blotting analysis

For the detection of EV (exosome) marker proteins and GFP, isolated EVs or lysate of CD63‐GFP‐HeLa cells was added to the SDS sample buffer. The boiled samples were separated via 10% SDS/PAGE, transferred onto polyvinylidene fluoride membranes (GE Healthcare, Pittsburgh, PA, USA), and treated with anti‐CD63 antibody (TS63; Abcam, Cambridge, UK), anti‐ALIX antibody (ab88388; Abcam), anti‐calnexin antibody (ab22595; Abcam), and anti‐GFP antibody‐ChIP grade (ab290; Abcam). A secondary antibody labeled with horseradish peroxidase [anti‐mouse IgG HRP NA931V (GE Healthcare) and goat anti‐rabbit IgG (ab6721, Abcam)] was used, and the immunoreactive species were detected using the Enhanced Chemiluminescence (ECL) Plus Western Blotting Detection System (GE Healthcare) with the Amersham Imager 600 (GE Healthcare) and iBright western blot imaging system (Thermo Fisher Scientific Inc.).

### Modification of EVs with EMCS‐R8 peptides

Synthesized EMCS‐R8 peptides (final 3.6 mm) diluted with H_2_O were added to a solution of EVs (36 μg) in PBS (total 90 μL) and incubated for 30 min at 25 °C. The attachment of FITC‐EMCS‐R8 to EVs was confirmed using a spectrofluorometer (FP‐6200, JASCO, Tokyo, Japan) after the removal of unattached peptides, which was accomplished by washing with PBS and filtration using Amicon Ultra‐centrifugal filters (100 KDa device, Merck Millipore, Billerica, MA, USA).

### Confocal microscopy (cellular uptake of EV samples)

A431 cells (each 2 × 10^5^ cells/2 mL) were seeded onto a 35‐mm glass dish (Iwaki) and incubated in MEM containing 10% FBS for 24 h at 37 °C under 5% CO_2_. After complete adhesion, the cells were then treated with each EV sample (200 μL per well) in MEM at different pH conditions (pH 5, 6, or 7), with or without 10% FBS. The cells were stained with Hoechst 33342 dye (Invitrogen; 5 μg·mL^−1^) for 15 min at 37 °C, prior to cell washing. The cells were then washed with fresh cell culture medium and analyzed using a FV1200 confocal laser scanning microscope (Olympus, Tokyo, Japan) equipped with a 40× objective without cell fixation.

### Confocal microscopy (CD63‐GFP stably expressing HeLa cells)

CD63‐GFP stably expressing HeLa cells (each 2 × 10^5^ cells/2 mL) were seeded onto a 35‐mm glass dish (Iwaki) and incubated in α‐MEM containing 10% FBS for 24 h at 37 °C under 5% CO_2_. After complete adhesion, the cells were treated with α‐MEM at different pH conditions, containing 10% FBS (pH 5 or 7; 2 mL per well) for 24 h at 37 °C under 5% CO_2_. The cells were then washed with fresh cell culture medium and analyzed using a FV1200 confocal laser scanning microscope (Olympus) equipped with a 40× objective without cell fixation.

### Flow cytometry (cellular uptake of EV samples)

A431 cells (each 1.4 × 10^5^ cells·mL^−1^) were plated onto a 24‐well microplate (Iwaki) and incubated in MEM containing 10% FBS for 24 h at 37 °C under 5% CO_2_. After complete adhesion, the cells were then treated with each EV sample (600 μL per well) in MEM at different pH conditions (pH 5, 6, or 7) with or without 10% FBS, prior to washing with PBS (triple washing, 200 μL). The cells were then treated with 0.01% trypsin at 37 °C for 10 min, prior to the addition of PBS (200 μL), and then centrifuged at 1500 r.p.m. (200 ***g***) for 5 min at 4 °C. After removal of the supernatant, the cells were washed with PBS (400 μL) and centrifuged at 1500 r.p.m. for 5 min at 4 °C. This washing cycle was repeated, and the cells were suspended in PBS (400 μL) and subjected to fluorescence analysis with a Guava easyCyte (Merck Millipore) flow cytometer using 488‐nm laser excitation and a 525‐nm emission filter. Live cells (10 000 cells per sample) for the detection of cellular fluorescence intensity were quantified based on forward‐scattering and side‐scattering analyses.

### Electron microscopy

Extracellular vesicles suspended in PBS (30 μg·mL^−1^) were dropped onto a carbon‐coated copper grid (250‐mesh), and then, the excess dispersion was removed with a filter paper. Then, 10 µL of 2% sodium phosphotungstate aqueous solution was dropped onto a grid and dried in a desiccator overnight, prior to imaging with a transmission electron microscope (TEM; JEM‐2000FEX II, JEOL, Tokyo, Japan) operated at 200 kV.

### Zeta potential, particle size, and number

The zeta potential and particle size of the EVs diluted in PBS (20 μg·mL^−1^) were determined using the zeta potential and particle size analyzer ELSZ‐DN2 (Otsuka Electronics, Osaka, Japan), according to the manufacturer's instructions. The particle number of EVs was measured by NanoSight LM10 with NTA2.3 Analytical software (Malvern Panalytical, Malvern, UK).

### Cell viability

A431 cells (each 1.4 × 10^5^ cells·mL^−1^) were plated onto a 24‐well microplate (Iwaki) and incubated in MEM containing 10% FBS for 24 h at 37 °C under 5% CO_2_. After complete adhesion, the cells were treated with each EV sample (600 μL·well^−1^) in MEM at different pH conditions (pH 5, 6, or 7), with or without 10% FBS, prior to washing with PBS (triple washing, 200 μL). The cells were then treated with 0.01% trypsin at 37 °C for 10 min, prior to the addition of PBS (200 μL). Following the addition of trypan blue (0.4% w/v; Gibco), the cell viability was determined using OneCell Counter (Bio Medical Science Inc., Tokyo, Japan) and TC20 automated cell counter (Bio‐Rad, Hercules, CA, USA).

### Statistical analyses

All statistical analyses were performed using the graphpad prism software (ver. 5.00; GraphPad, San Diego, CA, USA). For multiple comparison analyses, either one‐way analysis of variance (ANOVA) followed by Tukey's or Dunnett's *post hoc* test or two‐way ANOVA followed by Bonferroni's *post hoc* test was used. Differences were considered significant when the calculated *P*‐value was < 0.05.

## Results

### Increased expression of CD63‐GFP in HeLa cells under low pH condition

**Fig. 2:**
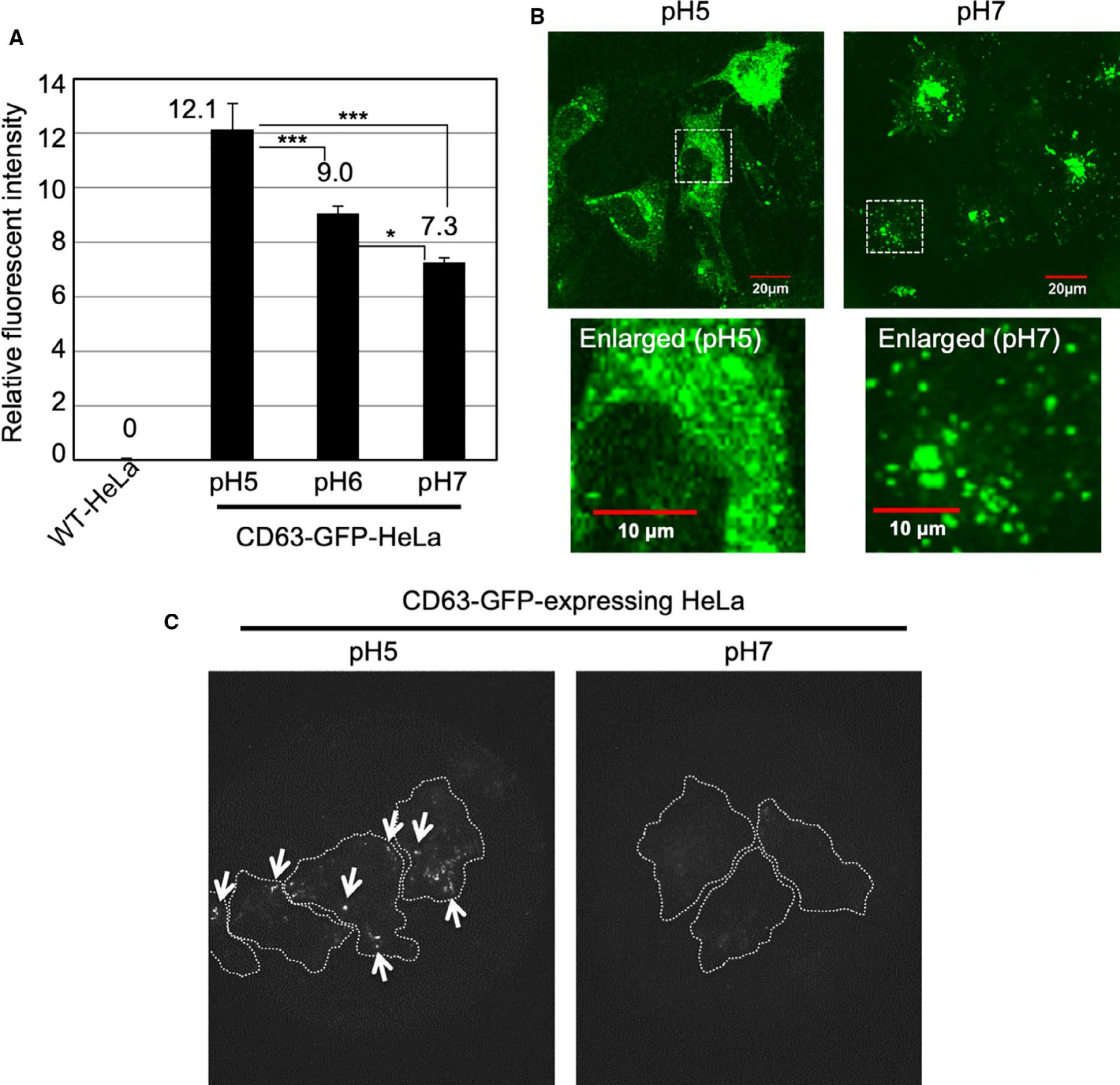
Effects of low pH cell culture condition on expression of CD63‐GFP fusion proteins. (A) Relative fluorescent intensity of CD63‐GFP fusion proteins stably expressing in HeLa cells cultured for 24 h at 37 °C in α‐MEM (pH 7, 6 or 5) with 10% FBS, analyzed using a flow cytometer (detection for 10 000 live cells). The data are expressed as the mean (± SD) of three experiments. Differences between groups were compared by one‐way ANOVA with Tukey's *post hoc* test. *P < 0.05, ***P < 0.001. (B) Confocal microscopic observation of CD63‐GFP fusion proteins stably expressing HeLa cells cultured for 24 h at 37 °C in α‐MEM (pH 7 or 5) with 10% FBS. Scale bar, 20 μm (enlarged pictures: 10 μm). (C) TIRF observation of CD63‐GFP fusion proteins stably expressing HeLa cells cultured for 24 h at 37 °C in α‐MEM (pH 7 or 5) with 10% FBS. Arrows show comparatively high fluorescent intensity of CD63‐GFP fusion protein expression in comparison with that at pH 7 cell culture condition. Dotted white lines show the cellular structures.

In this research, we used HeLa cells stably expressing CD63‐GFP fusion protein (CD63‐GFP‐HeLa) to achieve secretion of the CD63‐GFP‐expressing EVs (CD63‐GFP‐EVs) from the cells. This was used for assessing EV secretion and cellular uptake efficacy as previously reported [16, 21, 22]. CD63 is one of the exosomal tetraspanin proteins. Fluorescent protein‐fused CD63 is useful for the identification and cellular uptake/secretion tracking of the EVs, and the CD63‐GFP fusion protein‐expressing HeLa cells were prepared using antibiotic selection methods [16, 21, 22]. The CD63‐GFP‐HeLa cells were incubated in cell culture medium at different pH conditions (pH 5, 6, or 7) for 24 h at 37 °C under 5% CO_2_. Firstly, we examined the expression level of CD63‐GFP using a flow cytometer (Fig. 2A). Interestingly, we found that at low pH condition (pH 5 and 6), the fluorescent intensity of CD63‐GFP in the cells significantly increased in comparison with that in the normal pH condition (pH 7; Fig. 2A). Confocal microscopy analysis revealed that pH reduction significantly affected the intracellular distribution of GFP signals (Fig. 2B). In the case of pH 7 cell culture condition, GFP signals showed sparse locations in the cells, as typical distribution previously reported [16, 21, 22] (Fig. 2B). However, low pH cell culture conditions (pH 5) significantly affected the distribution of CD63, and dispersed‐GFP signals in the cytosol were observed in time‐dependent manner (Fig. 2B, Fig. S1). In the experimental condition, although the low pH cell culture condition significantly decreased the proliferation rate of CD63‐GFP‐HeLa cells, the number of dead cells was very low and changes in cell morphology were not observed even in low pH condition (Fig. S2). These results suggest that pH reduction increased the expression level of CD63‐GFP in the HeLa cells and possibly affected the distribution patterns of MVBs in which EVs are generated and/or their intracellular traffic routes for EV secretion.

### Low pH increases expressed CD63‐GFP near plasma membranes

Next, we examined the amount of expressed CD63‐GFP near the plasma membranes using total internal reflection fluorescence (TIRF) microscopy (Fig. 2C). TIRF microscopy induces evanescent wave in a limited specimen region and is used for the effective visualization of the contact area between the specimen (basal plasma membrane surface) and a glass‐base cell culture dish. For secretion of the EVs, membrane fusion of MVBs and plasma membranes are indispensable [2, 23]. Low pH resulted in abundance of CD63‐GFP near the basal plasma membranes in comparison with that at pH 7 (Fig. 2C). The results suggest that the low pH cell culture condition possibly affects the efficacy of EV secretion because of their enrichment near plasma membranes.

### Effects of low pH on the secretion of EVs per cells

**Fig. 3:**
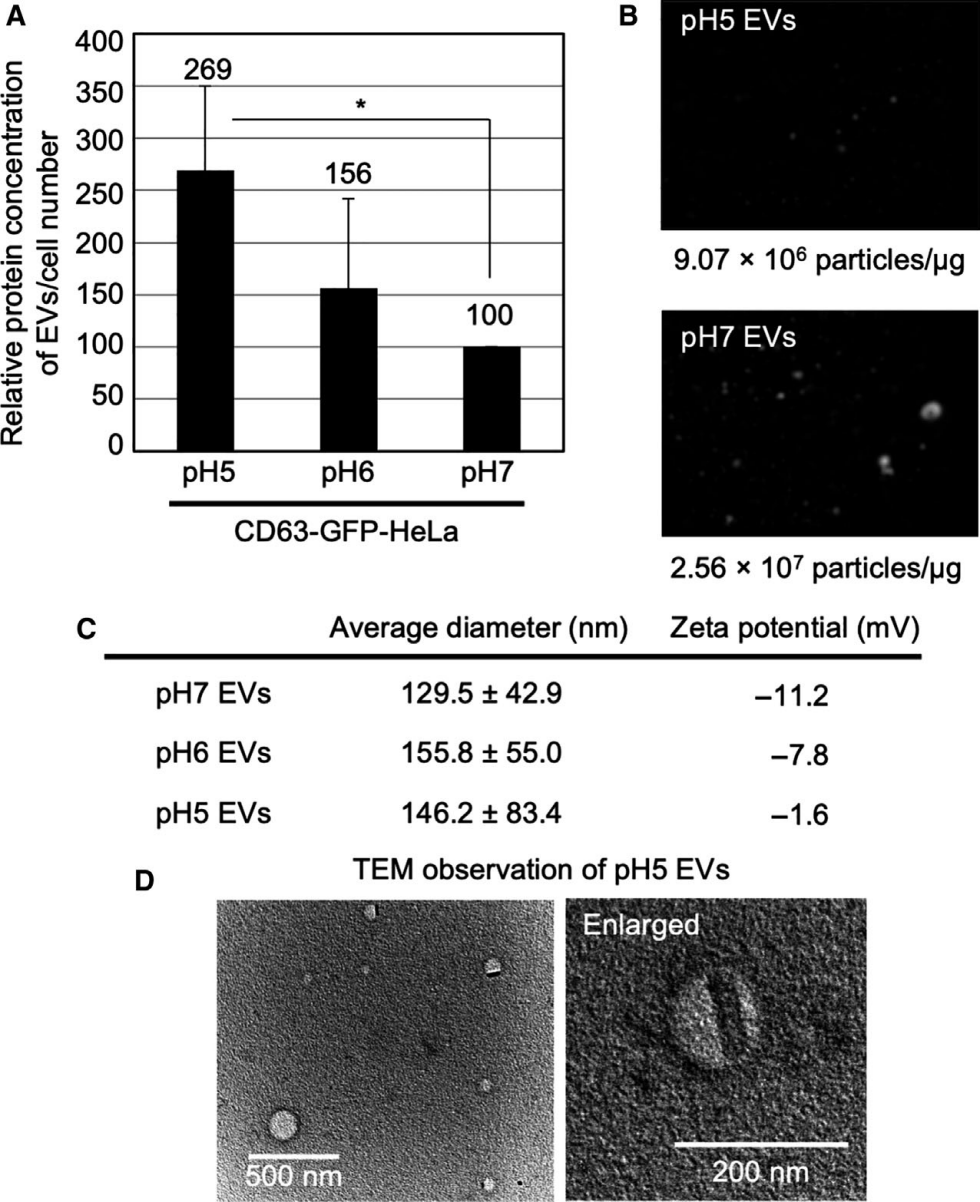
Increased protein content of secreted EVs under low pH cell culture condition. (A) Relative protein concentration of EVs/cell number under different pH conditions (pH 5, 6, or 7) cell culture condition. EVs secreted from CD63‐GFP‐HeLa cells (48 h, 37 °C) were analyzed by BCA protein assay, and each protein concentration was divided by the cell number. The data are expressed as the mean (± SD) of three experiments. Differences between groups were compared by one‐way ANOVA with Dunnett's *post hoc* test. *P < 0.05. (B) Particle images and number of secreted EVs were detected by NanoSight. (C) Average diameter and zeta potential of the isolated CD63‐GFP‐EVs secreted from CD63‐GFP‐HeLa under same cell culture condition as that in (A). (D) TEM observation of CD63‐GFP‐EVs secreted by CD63‐GFP‐HeLa cells under pH 5 condition of (A). Scale bar: 500 nm (left) and 200 nm (right, enlarged).

We assessed the efficacy of EV secretion from CD63‐GFP‐HeLa cells in cell culture at different pH conditions (pH 5, 6, or 7; Fig. 3A). CD63‐GFP‐HeLa cells were incubated in each pH cell culture medium with EV‐free 10% FBS for 48 h at 37 °C, and the cell culture medium was collected for the isolation of secreted EVs using ultracentrifugation method, as described in Materials and methods (pH 5 EVs: secreted from the cells incubated in pH 5 cell culture medium; pH 6 EVs: secreted from the cells incubated in pH 6 cell culture medium; pH 7 EVs: secreted from the cells incubated in pH 7 cell culture medium). Figure 3A shows the relative protein concentration of secreted EVs (per cell number), and the pH 5 cell culture condition significantly increased the secretion amount of EV proteins in comparison with that in pH 7 cell culture condition. In time‐dependent analysis, the secretion amount of EV proteins in pH 7 cell culture condition was higher than that in the pH 5 cell culture condition in a short period (24 h); however, reversal results were interestingly confirmed in a long period (48 h; Fig. S3A). The average concentration of EVs [particles/EV protein (μg)] analyzed by NanoSight showed 9.07 × 10^6^ particles·μg^−1^ (pH 5 EVs) and 2.56 × 10^7^ particles·μg^−1^ (pH 7 EVs; Fig. 3B), suggesting that the low pH possibly increases EV protein concentration per EV particle in 48‐h condition. Tetraspanin CD63 and ALIX, which are EV marker protein, were detected by western blot analysis (Fig. S4A,B). Calnexin, which is a negative EV marker, was not expressed in CD63‐GFP‐EVs although the calnexin expression was confirmed in CD63‐GFP‐HeLa (Fig. S4C). As mentioned above, low pH condition significantly affected the intracellular distribution of GFP signals with cytosolic diffusion (Fig. 2B). Western blot analysis confirmed that pH reduction did not induce degradation of CD63‐GFP in the cells (Fig. S4D). The average diameter of the isolated CD63‐GFP‐EVs secreted from CD63‐GFP‐HeLa cells grown at pH 7 with EV‐free 10% FBS for 48 h at 37 °C was 129.5 ± 42.9 nm, and the zeta potential of the isolated EVs, determined using a particle size and zeta potential analyzer, was shown to be −11.2 mV (Fig. 3C). The average diameter of the isolated EVs secreted from CD63‐GFP‐HeLa cells cultured at pH 6 or 5 was 155.8 ± 55.0 nm (pH 6 EVs) or 146.2 ± 83.4 nm (pH 5 EVs), suggesting a negligible effect on the average diameter by the low pH condition (Fig. 3C). However, the zeta potential of the isolated EVs secreted from CD63‐GFP‐HeLa cells cultured at pH 6 or 5 was shown to be −7.8 mV (pH 6 EVs) and −1.6 mV (pH 5 EVs; Fig. 3C), suggesting that the low pH cell culture condition significantly affects the zeta potentials of secreted EVs. Figure 3D shows the transmission electron microscopy (TEM) observations of the isolated CD63‐GFP‐EVs secreted from CD63‐GFP‐HeLa cells in the cell culture medium at pH 5 with EV‐free 10% FBS for 48 h at 37 °C; they have a vesicular structure, suggesting almost no effect of the low pH cell culture condition on their EV morphology (Fig. 3D).

### Increased cellular uptake efficacy (serum‐starved condition) of isolated EVs secreted under low pH cell culture condition

**Fig. 4:**
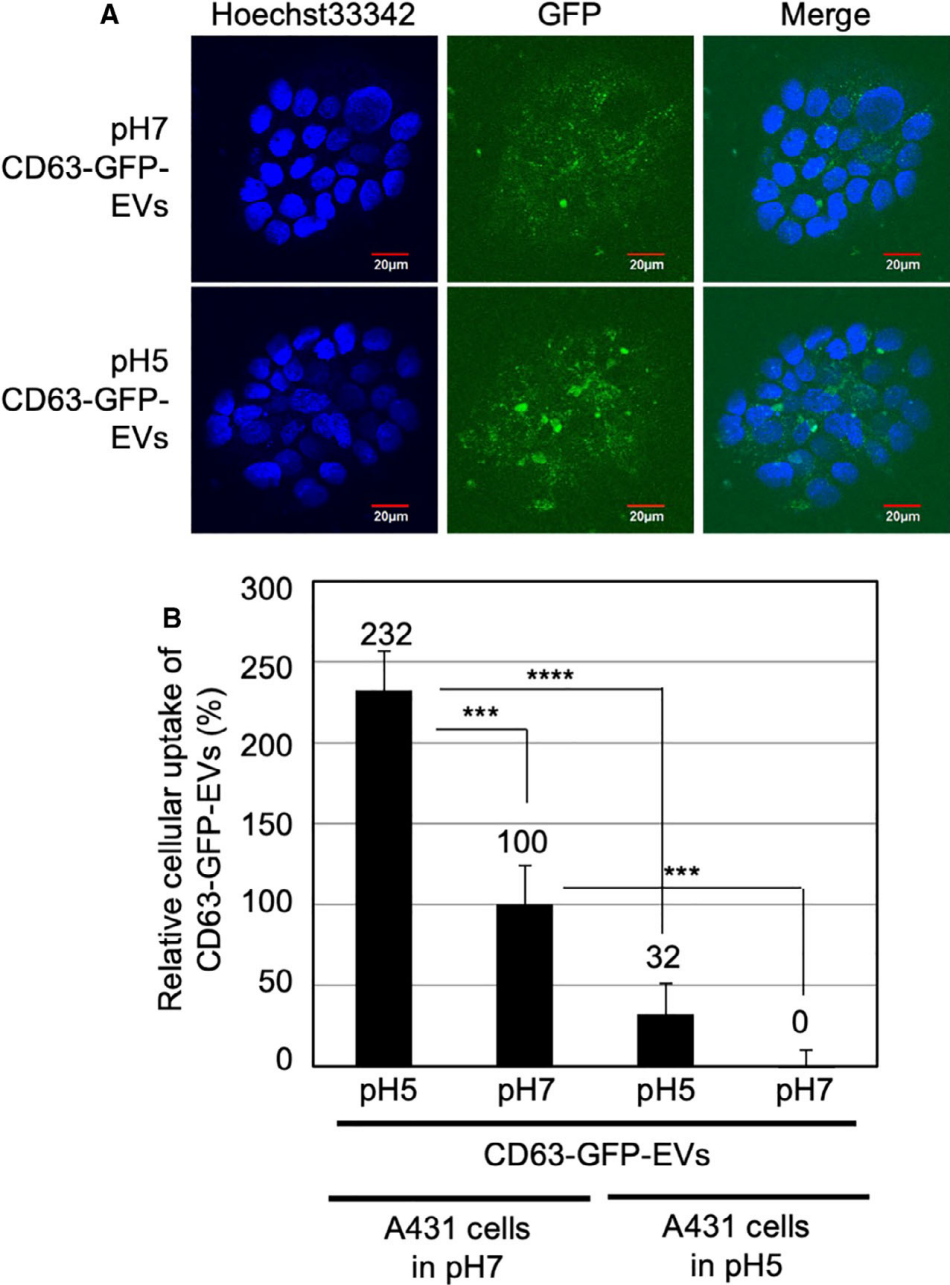
Increased cellular uptake (serum‐starved condition) of isolated EVs secreted under low pH cell culture condition. (A) Confocal microscopic observation of A431 cells treated with isolated CD63‐GFP‐EVs [20 μg·mL^−1^ for 48 h at 37 °C in MEM (pH 7) without FBS], secreted under pH 7 or pH 5 cell culture condition. Blue signals, Hoechst 33342 for nuclear staining; green signals, CD63‐GFP‐EVs. Scale bar, 20 μm. (B) The relative cellular uptake of isolated CD63‐GFP‐EVs [20 μg·mL^−1^ for 48 h at 37 °C in MEM (pH 7 or 5) without FBS] secreted under pH 7 or pH 5 cell culture condition, analyzed using a flow cytometer (detection for 10 000 live cells). The data are expressed as the mean (± SD) of three experiments. Differences between groups were compared by two‐way ANOVA followed by Bonferroni's *post hoc* test ***P < 0.001, ****P < 0.0001.

Next, because zeta potentials of EV membranes possibly affect the cellular EV uptake, we examined the cellular uptake efficacy of the isolated CD63‐GFP‐EVs. Generally, plasma membranes are negatively charged, and zeta potentials of nanomaterials (e.g., liposomes) have been shown to affect their internalization by cells. A431 cells were treated with each isolated CD63‐GFP‐EVs (20 μg·mL^−1^) for 48 h at 37 °C in the cell culture medium (MEM) without FBS (serum‐starved condition), prior to the cell nuclear staining with Hoechst 33342 and confocal laser microscopic observation (Fig. 4A). The CD63‐GFP‐EVs secreted in pH 5 cell culture condition showed comparatively stronger intensity of fluorescent signals upon internalization in comparison with that of CD63‐GFP‐EVs secreted in pH 7 cell culture condition (Fig. 4A). Flow cytometry analysis of internalized CD63‐GFP pH 5 EVs also resulted in ca. twofold higher uptake by the A431 cells cultured in pH 7 cell culture medium than that in pH 7 EVs (Fig. 4B). In time‐dependent analysis of the cellular uptake, 24‐h treatment showed the highest cellular uptake of the CD63‐GFP‐EVs secreted in pH 5 cell culture condition (Fig. S3B). In the cellular EV uptake experiment (pH 7 cell culture medium condition for A431 cells), cell viability was not affected by the treatment of pH 5 or pH 7 EVs (Fig. S5). In the case of A431 cells cultured in pH 5 cell culture medium, ca. 50% of the cells were dead (Fig. S5). However, the CD63‐GFP‐EVs secreted in pH 5 cell culture condition also showed higher internalization of EVs into living cells than that of CD63‐GFP‐EVs secreted in pH 7 cell culture condition (Fig. 4B). These results suggest that the EVs secreted in pH 5 cell culture condition have an ability to enhance their cellular uptake efficacy.

### Effects of serum present in cell culture medium and modification of arginine‐rich cell‐penetrating peptides on the cellular uptake efficacy of isolated EVs

**Fig. 5:**
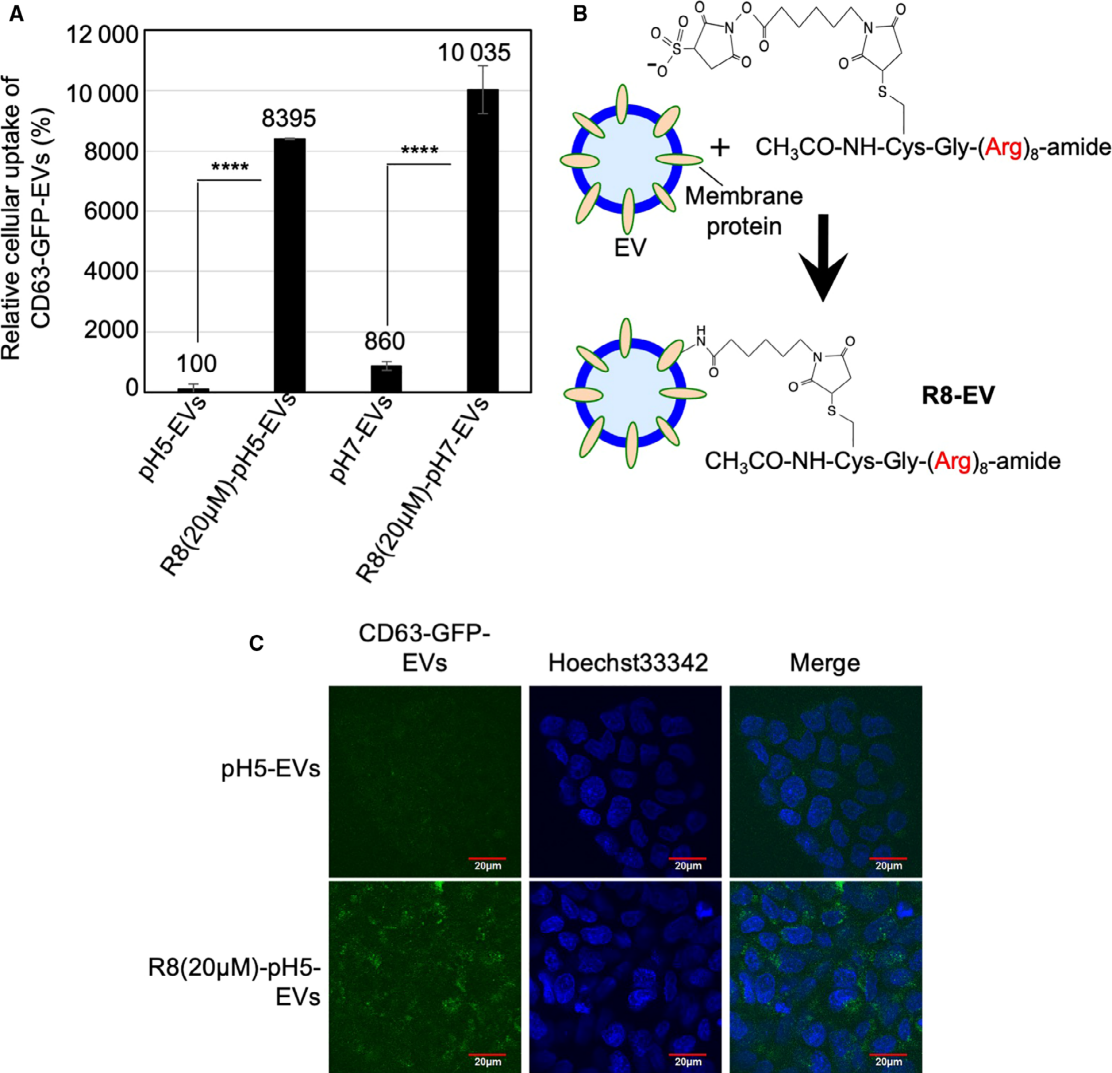
Effect of serum and modification of octaarginine (R8) peptides on cellular EV uptake. (A) The relative cellular uptake (A431 cells) of isolated CD63‐GFP‐EVs [20 μg·mL^−1^ for 24 h at 37 °C in MEM (pH 7) with 10% FBS] secreted under pH 7 or pH 5 cell culture condition, analyzed using a flow cytometer (detection for 10 000 live cells). The data are expressed as the mean (± SD) of three experiments. Differences between groups were compared by two‐way ANOVA followed by Bonferroni's *post hoc* test. ****P < 0.0001. (B) Modification of R8 peptides on exosomal membrane via suffo‐EMCS linker. (C) Confocal microscopic observation of A431 cells treated with isolated CD63‐GFP‐EVs [20 μg·mL^−1^ for 24 h at 37 °C in MEM (pH 7) with FBS] secreted under pH 5 cell culture condition, with or without R8 peptide modification on EV membrane. Blue signals, Hoechst 33342 for nuclear staining; green signals, CD63‐GFP‐EVs. Scale bar, 20 μm.

Presence of serum in the media affects the cellular uptake efficacy of carrier molecules such as cationic liposomes because of their interaction with serum contents [32]. Hence, we examined the effects of serum on the cellular uptake of EVs. A431 cells were treated with isolated CD63‐GFP‐EVs (20 μg·mL^−1^) for 24 h at 37 °C in the cell culture medium (MEM) with 10% FBS, prior to flow cytometry analysis, for detecting the CD63‐GFP‐EV internalization (Fig. 5A). Interestingly, in the case of serum‐containing condition, flow cytometry analysis of internalized CD63‐GFP‐EVs secreted in pH 5 cell culture condition resulted in ca. 8.6‐fold lower than that of pH 7 EVs by the A431 cells cultured in pH 7 cell culture medium (Fig. 5A).

Arginine‐rich cell‐penetrating peptides (CPPs), including HIV‐1 Tat (48–60) and oligoarginines, have been shown to be efficiently internalized by various types of cells [33]. Therefore, the CPPs have been widely applied as intracellular delivery carriers to increase cellular uptake of biofunctional molecules, including proteins, peptides, nucleic acids, and artificial polymers [33]. The arginine‐rich CPPs can effectively induce macropinocytosis (accompanied by actin reorganization, ruffling of plasma membrane, and engulfment of large volumes of extracellular fluid), leading to efficient cellular uptake of the CPPs [28, 29, 33, 34]. The macropinocytosis pathway is also important for the cellular uptake of EVs. We earlier reported that the activation of cancer‐related receptors (e.g., epidermal growth factor receptor) and the expression of oncogenic K‐Ras can enhance macropinocytosis, resulting in enhanced cellular uptake of EVs [16]. In addition, we successfully developed the techniques for the modification of EV membranes with octaarginine (R8) peptide, which is one of the representative arginine‐rich CPPs, to attain increased cellular EV uptake [21]. In this research, we examined the effects of CPP modification on cellular uptake of the EVs secreted from cell culture at different pH conditions. The EV membranes were modified with R8 peptides, which was achieved by mixing the EVs with R8‐EMCS (*N*‐ε‐maleimidocaproyl‐oxysuccinimide ester), an amine‐to‐sulfhydryl crosslinker (Fig. 5B). Fluorescently labeled peptides (FITC‐R8‐EMCS) were used to assess the binding of the R8 peptides to the EV membrane using a spectrofluorometer, and the method resulted in the binding of FITC‐R8‐EMCS (2.5 μm for EV secreted in pH 5 condition and 2.9 μm for EV secreted in pH 7 condition) to each EV (20 μg·mL^−1^). The A431 cells were treated with the isolated CD63‐GFP‐EVs [20 μg·mL^−1^ for 24 h at 37 °C in MEM (pH 7) with 10% FBS] secreted under pH 7 or pH 5 cell culture condition, analyzed using a flow cytometer or confocal microscopic observation (Fig. 5A,C). Cellular uptake of each EV was significantly enhanced, and the CPP modification on EV membranes resulted in almost similar cellular uptake efficacies of EVs secreted in cell culture at different pH conditions (Fig. 5A,C). In this experimental condition, the CPP modification did not induce any cytotoxicity (Fig. S6). These results suggest that the arginine‐rich CPPs are suitable enhancers for uptake of isolated EVs, and the membrane composition of EVs might possibly be one of the mechanisms through which pH of the medium affects the cellular uptake of EVs.

## Discussion

In this research, we found that the reducing the pH of cell culture medium significantly increased the expression level of GFP fused with an EV (exosome) marker protein, CD63 [1, 3] in the HeLa cells (Fig. 2A), and affected their intracellular localization (Fig. 2B). In addition, higher number of dot‐like fluorescent signals near the basal plasma membranes of cells in the cell culture medium with low pH (pH 5) was observed than those in the basal plasma membranes of cells grown at pH 7 cell culture condition (Fig. 2C). As a related secretion system, the lysosomal protease cathepsin B is secreted from a variety of human tumors. Rozhin and coworkers reported that an acidic pericellular pH (pH 6.5) induced the translocation of lysosomes, and redistribution of cathepsin B vesicles toward the cell periphery [35]. Translocation and secretion of cathepsin B were dependent on a functional microtubular system, redistribution of cathepsin B vesicles toward the cell surface was induced by acidic pH, and the acidic pH‐induced secretion of hyperactive cathepsin B was inhibited by microtubule inhibitors [35]. The findings regarding the cathepsin B resemble our results; however, their detailed molecular mechanisms are still unknown. In addition, there were established EV techniques including the anti‐GFP fluobody expressing system to assess membrane fusion of EVs and endosomes [36], EV cargo release technique using proteases [37], and intracellular visualization technique using Renilla luciferase [38], and the experimental systems will contribute for not only elucidation of CD63‐GFP cytosolic diffusion in the low pH condition but also cellular uptake, membrane fusion, and cytosolic release of EV contents.

Differences between intracellular pH (pHi) and extracellular pH (pHo) exist in normal mammalian tissues, with pHi being approximately 0.3 units lower than that of pHo [39]. However, in tumor cells, this gradient of pH is normally collapsed, or very often, reversed (i.e., tumor cells often create an acidic extracellular environment, but display basic intracellular pH as a consequence of increased metabolic activity) [39]. Tumor cells might raise their set point for pHi by increasing the export of H^+^ from the intracellular compartment [39, 40]. Increased H^+^ export could permit faster overall production of acid by tumor metabolism and lead indirectly to increased extracellular acidity [39, 40]. The increased H^+^ export could be achieved via activation of the mitogen‐sensitive Na^+^/H^+^ exchanger, or via increased functional expression in the plasma membrane of the H^+^‐pumping ATPases [39, 40]. Functional expression of H^+^‐pumping ATPases has been measured on the cell surface of some tumor cells, particularly those with a higher pHi, and it is associated with greater ATP turnover, increased glycolysis, and decreased protein degradation, all of which are features of the tumor metabolic phenotype [39, 40]. Addition of ligands that cross‐link mitogen receptors on the cell surface induces a rapid increase in pHi of up to 0.2 units [41, 42]. Therefore, our experimental results might contribute to understanding the EV‐based cell–cell communication found in cancer cells. Bumke and coworkers demonstrated a broad range analysis of the patterns of gene expression in normal human dermal fibroblasts at different pH values, and the expression of 2068 genes (out of 12 565) was modulated by more than twofold after the shift of the cell culture medium pH to a more acidic value (e.g., stanniocalcin 1 is a remarkable example of a strongly up‐regulated gene) [39]. Sixty‐seven out of 2068 genes showed a modulation of expression greater than fivefold at 72 h [39]. Genes that displayed a modulated pattern of expression included the cell cycle regulators (consistent with the observation that acidic pH abolished the growth of fibroblasts in culture) and relevant extracellular matrix components [39]. Certain genes encoding for growth factor receptors show an increase in their expression level at acidic pHo, such as the platelet‐derived growth factor receptor‐like coding gene and a growth factor‐inducible nuclear protein N10 [39]. In our research, acidic pH of extracellular fluid induced the enhanced expression of EV proteins, and these former findings of increased expression of genes in acidic cell culture condition might relate to the case of EV protein expression; however, further elucidation of the mechanisms would be required.

Low pH cell culture condition highly affected the secretion quantity of EVs. Although the protein concentration in the EVs was shown to be increased in the experimental condition of low pH cell culture (48 hr), the number of EVs was reduced by the pH reduction (Fig. 3A,B). EVs are generated in MVB in cells as intraluminal vesicles by inward budding of the membrane of MVB, and the MVBs are fused with the plasma membrane prior to the release of EVs to the extracellular space, in an exocytic manner [2, 23]. As described in Introduction section, ESCRT‐dependent pathway (multiprotein complexes of ESCRT‐0, ESCRT‐I, ESCRT‐II, and ESCRT‐III) has been pointed out to play crucial roles in the mechanism of EV biogenesis [24, 25, 26]. In addition, the budding and/or release of EVs have been shown to be controlled by lipids, including sphingolipid ceramide [24] or sphingosine 1 phosphate within the ESCRT‐independent pathway [26]. Acidic pH of extracellular fluid might affect the inward budding of the membrane of MVB even with enhanced expression of EV proteins and their release to the extracellular space; however, the detailed mechanisms are unknown and should be further studied. In addition, in time‐dependent analysis, the secretion amount of EV proteins in pH 7 cell culture condition showed higher than that of the pH 5 cell culture condition in a short period (24 h); however, reversal results were interestingly confirmed in a long period (48 h; Fig. S3A). In Fig. 5, cellular uptake efficacy of the EVs secreted in pH 7 showed higher than that of the EVs secreted in pH 5 in serum‐containing condition might affect the isolation efficacy of the EVs. Possible negative feedback to EV secretion is also conceivable. In addition, stability of the EV secreted in each pH condition is considered to be affected, and the detailed mechanisms should be further studied in the complicated points. The time‐dependent manner of EV secretion and cellular uptake will contribute to understanding cell‐to‐cell communication mechanisms and development of EV‐based intracellular delivery system.

Cellular EV uptake efficacy was highly affected by the EV secretion condition prior to the EV collection (Figs 4 and 5). For avoidance of serum influence over cellular EV uptake, we firstly examined their cellular uptake in serum‐starved experimental condition (Fig. 4). The EVs derived from HeLa cells cultured in low pH condition resulted in higher cellular uptake efficacy in comparison with that of neutral pH condition (Fig. 4). The zeta potential of EV membranes derived from HeLa cells cultured in low pH condition changed to −1.6 mV from −11.2 mV (pH 7 cell culture condition; Fig. 3C). The increased zeta potential of EV membranes might be able to increase the interplay with plasma membranes of targeted cells because of the negatively charged cell membranes (e.g., zeta potential of HeLa cells: −19.4 mV) [43], leading to enhanced cellular EV uptake (Fig. 4). However, the presence of serum (10% FBS containing media) resulted in the opposite effect of cellular uptake of EVs, with the EVs derived in pH 7 cell culture condition having higher ability of cellular uptake than that of the EVs derived in pH 5 (Fig. 5A). One of the possible mechanisms might be the interaction of serum contents with the EVs similar to that of liposomes [32], leading to covering the serum contents on EV membranes and to reduction in their cellular uptake efficacy.

Arginine‐rich CPPs including the R8 peptide have high abilities for macropinocytosis induction and effective cellular uptake [28, 29, 33, 34]. The CPPs efficiently accumulate on plasma membranes via glycosaminoglycans, leading to clustering of proteoglycans such as syndecan‐4 and binding of PKCα to the clustered proteoglycans for signal transduction to induce macropinocytosis [28, 29, 33, 34]. The isolated EVs when coated with R8‐EMCS (Fig. 5B) resulted in enhanced cellular uptake to levels between the EVs derived in pH 5 and pH 7 experimental conditions (Fig. 5A,C). The results suggest not only the usefulness of the CPP modification on EV membranes to enhance cellular EV uptake, but also that membrane components of the EV might be essential factors for deciding cellular uptake efficacy of the EVs, affected by environmental pH condition prior to the EV collection and isolation.

In conclusion, we found in this research that low pH cell culture condition affects various parameters of the secretion of the EVs, including EV protein content, EV particle number, and EV zeta potential, leading to an effect on their cellular uptake. The findings will contribute to understanding cell–cell communication based on EVs, which can be affected by microenvironments of cells during disease progression, and to develop intracellular delivery of biologically functional molecules on the basis of EVs.

## Conflict of interest

The authors declare no conflict of interest.

## Author contributions

IN designed the study, performed the experiments (peptide synthesis, confocal microscopy, zeta potential, particle size, and number), and wrote the manuscript. NU performed the experiments (peptide synthesis, peptide modification on EVs, confocal microscopy, flow cytometry, zeta potential, particle size, and number). MM performed the experiments (confocal microscopy, flow cytometry, zeta potential, particle size, and number). KN performed the experiments (peptide synthesis, peptide modification on EVs). MH performed the experiments (EV isolation, western blot). MO performed the experiments (flow cytometry and western blot). TT performed the experiments (EV number, particle size). AS performed the experiments (western blot). NBK performed the experiments (preparation of CD63‐GFP stably expressing cells). TH performed the experiments (electron microscopy). TT‐N designed the study and conducted statistical analysis. EY performed the experiments (electron microscopy). IF designed the study and assessed mass spectrometry data. SF designed the study and assessed cellular uptake. TY designed the study and performed the experiments (preparation of CD63‐GFP stably expressing cells).

## Supporting information


**Fig. S1.** Effects of low pH cell culture condition on expression of CD63‐GFP fusion proteins.
**Fig. S2.** Viability of cells stably expressing the CD63‐GFP fusion protein.
**Fig. S3.** Protein content of secreted EVs under low pH cell culture condition and their cellular uptake.
**Fig. S4.** Western blot analyses of CD63‐GFP‐EVs and CD63‐GFP‐HeLa cells.
**Fig. S5.** Cell viability under cellular EV uptake experiments in serum‐starved condition.
**Fig. S6.** Cell viability under cellular EV uptake experiments in 10% FBS containing condition.

## Data Availability

Data will be available from the corresponding author upon reasonable request.
