# Supplementary material for: Environmental pH stress influences cellular secretion and uptake of extracellular vesicles
Source: FEBS Open Bio. 2021 Feb 18;11(3):753–67. doi: 10.1002/2211-5463.13107 (PMC7931216; doi:10.1002/2211-5463.13107)
Supplement: Supplementary file 1 — Fig. S1. Effects of low pH cell culture condition on expression of CD63‐GFP fusion proteins. Fig. S2. Viability of cells stably expressing the CD63‐GFP fusion protein. Fig. S3. Protein content of secreted EVs under low pH cell culture condition and their cellular uptake. Fig. S4. Western blot analyses of CD63‐GFP‐EVs and CD63‐GFP‐HeLa cells. Fig. S5. Cell viability under cellular EV uptake experiments in serum‐starved condition. Fig. S6. Cell viability under cellular EV uptake experiments in 10% FBS containing condition. [file FEB4-11-753-s001.pdf]

FEBS Open Bio (Research Article)

Supplementary Information

Title:

Environmental pH stress influences cellular secretion and uptake of extracellular vesicles

Authors:

Ikuhiko Nakase[1,2,*], Natsumi Ueno[1,2], Mie Matsuzawa[2], Kosuke Noguchi[1,2], Mami Hirano[1,2], Mika Omura[1,2], Tomoya Takenaka[1,2], Ayaka Sugiyama[1,2], Nahoko Bailey Kobayashi[3,4], Takuya Hashimoto[5], Tomoka Takatani-Nakase[6,7], Eiji Yuba[5], Ikuo Fujii[1], Shiroh Futaki[8], and Tetsuhiko Yoshida[3,4]

Affiliations:

[1]Graduate School of Science, Osaka Prefecture University, 1-1, Gakuen-cho, Naka-ku, Sakai, Osaka 599-8531, Japan

[2]NanoSquare Research Institute, Osaka Prefecture University, 1-2, Gakuen-cho, Naka-ku, Sakai, Osaka 599-8531, Japan

[3]Keio University School of Medicine, 2, Okubo, Tsukuba, Ibaraki 300-2611, Japan

[4]Institute for Advanced Sciences, Toagosei Co., Ltd., 2, Okubo, Tsukuba, Ibaraki 300-2611, Japan

[5]Department of Applied Chemistry, Graduate School of Engineering, Osaka Prefecture University, 1-1, Gakuen-cho, Naka-ku, Sakai-shi, Osaka 599-8531, Japan

[6]Department of Pharmaceutics, School of Pharmacy and Pharmaceutical Sciences, Mukogawa Women's University, 11-68, Koshien Kyuban-cho, Nishinomiya, Hyogo 663-8179, Japan

[7]Institutre for Bioscience, Mukogawa Women's University, 11-68, Koshien Kyuban-cho, Nishinomiya, Hyogo 663-8179, Japan

[8]Institute for Chemical Research, Kyoto University, Gokasho, Uji, Kyoto 611-0011, Japan

***Correspondence to*:** Ikuhiko Nakase, Graduate School of Science, Osaka Prefecture University, 1-1, Gakuen-cho, Naka-ku, Sakai, Osaka 599-8531, Japan. Tel: +81 722549895, Fax: +81 722549895, e-mail: i-nakase@21c.osakafu-u.ac.jp.

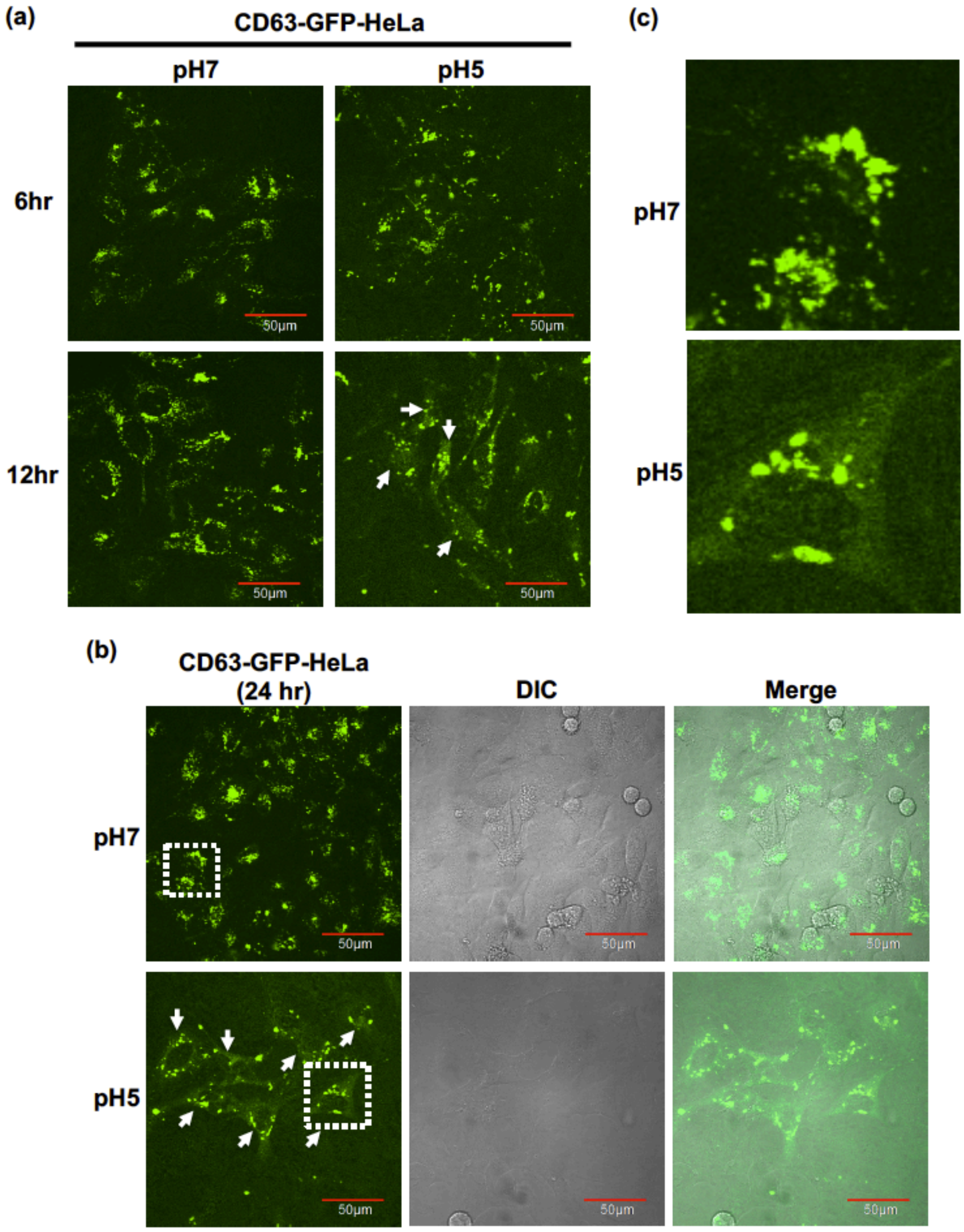

Supplementary Figure S1. Effects of low pH cell culture condition on expression of CD63-GFP fusion proteins. (**a**, **b**) Confocal microscopic observation of CD63-GFP fusion proteins stably expressing HeLa cells cultured for 6, 12 (**a**), and 24 h (**b**) at 37°C in α-MEM (pH 7 or 5) with 10% FBS. Scale bar, 50 μm. Arrows show typical cells with diffused GFP signals in cytosol. (**c**) Enlarged pictures of (**b**) (areas within the white dot squares).

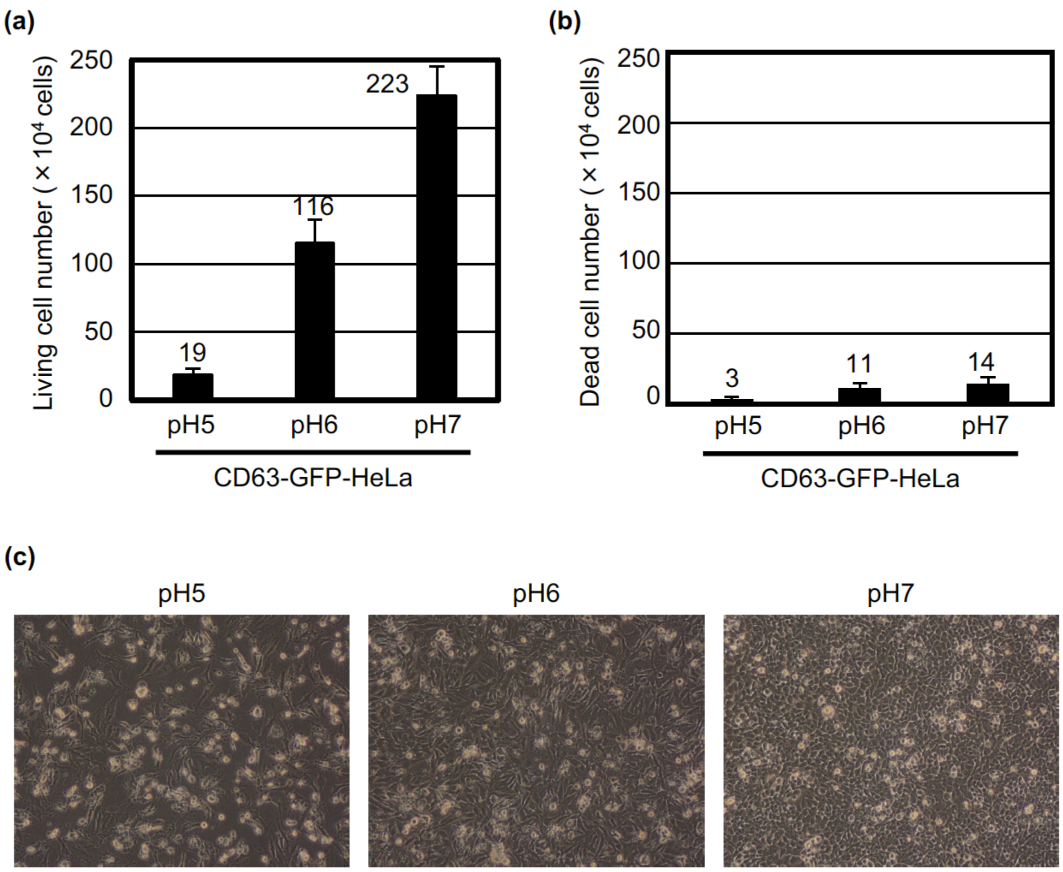

Supplementary Figure S2. Viability of cells stably expressing the CD63-GFP fusion protein. (**a**, **b**) Living (**a**) or dead (**b**) cell counting assay after culture of HeLa cells stably expressing the CD63-GFP fusion protein for 24 h at 37°C in α-MEM (pH 5, 6, or 7) with 10% FBS in trypan blue stain methods. The data are expressed as the mean (± SD) of three experiments. (**c**) Microscopic observation of CD63-GFP fusion proteins stably expressing HeLa cells in α-MEM (pH 5, 6, or 7) with 10% FBS for 48 h at 37°C.

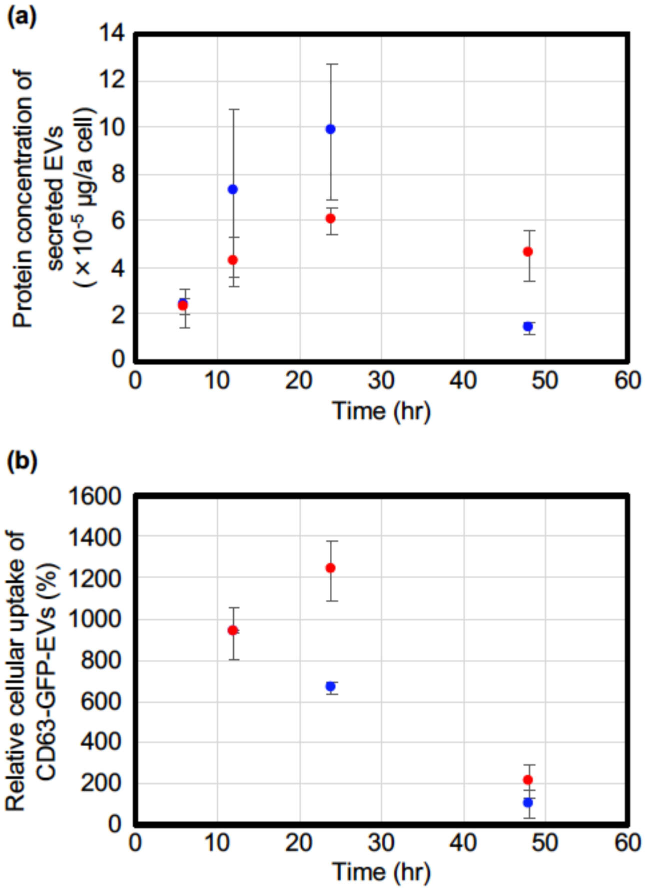

Supplementary Figure S3. Protein content of secreted EVs under low pH cell culture condition and their cellular uptake. (**a**) Relative protein concentration of EVs/cell number under different pH (pH 5 (red) or 7 (blue)) cell culture condition. EVs secreted from CD63-GFP-HeLa cells (6, 12, 24, or 48 h, 37°C) were analyzed by BCA protein assay, and each protein concentration was divided by the cell number. The data are expressed as the mean (± SD) of three experiments. (**b**) Cellular uptake (serum-starved condition) of isolated EVs. The relative cellular uptake of isolated CD63-GFP-EVs (20 µg/mL for 12, 24, or 48 h at 37°C in MEM (pH 7) without FBS) secreted under pH 7 (blue) or pH 5 (red) cell culture condition, analyzed using a flow cytometer (detection for 10,000 live cells). The data are expressed as the mean (± SD) of three experiments.

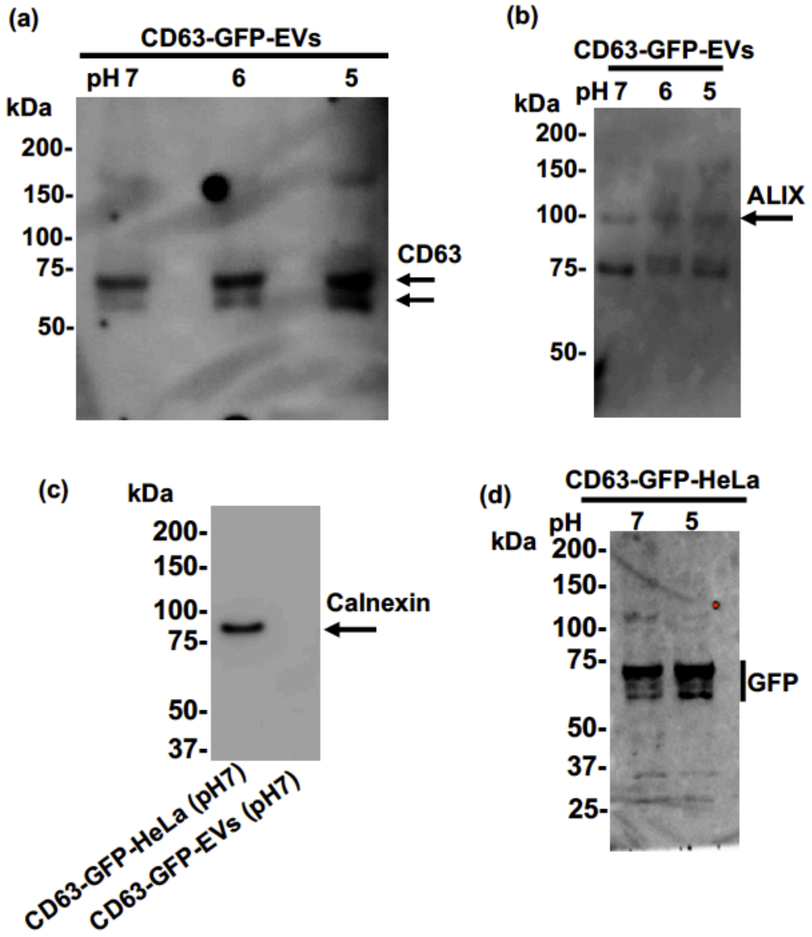

Supplementary Figure S4. Western blot analyses of CD63-GFP-EVs and CD63-GFP-HeLa cells. Western blot analyses (anti-CD63 (**a**), anti-ALIX (**b**), anti-calnexin (**c**), and anti-GFP (**d**)) showing the EVs secreted from CD63-GFP-HeLa under pH5, 6, or 7 condition (48 hr) (**a**, **b**), lysate of CD63-GFP-HeLa (pH7, 48 hr) and CD63-GFP-EVs (pH7, 48 hr) (**c**), and lysate of CD63-GFP-HeLa (pH7 or pH5, 48 hr) (**d**).

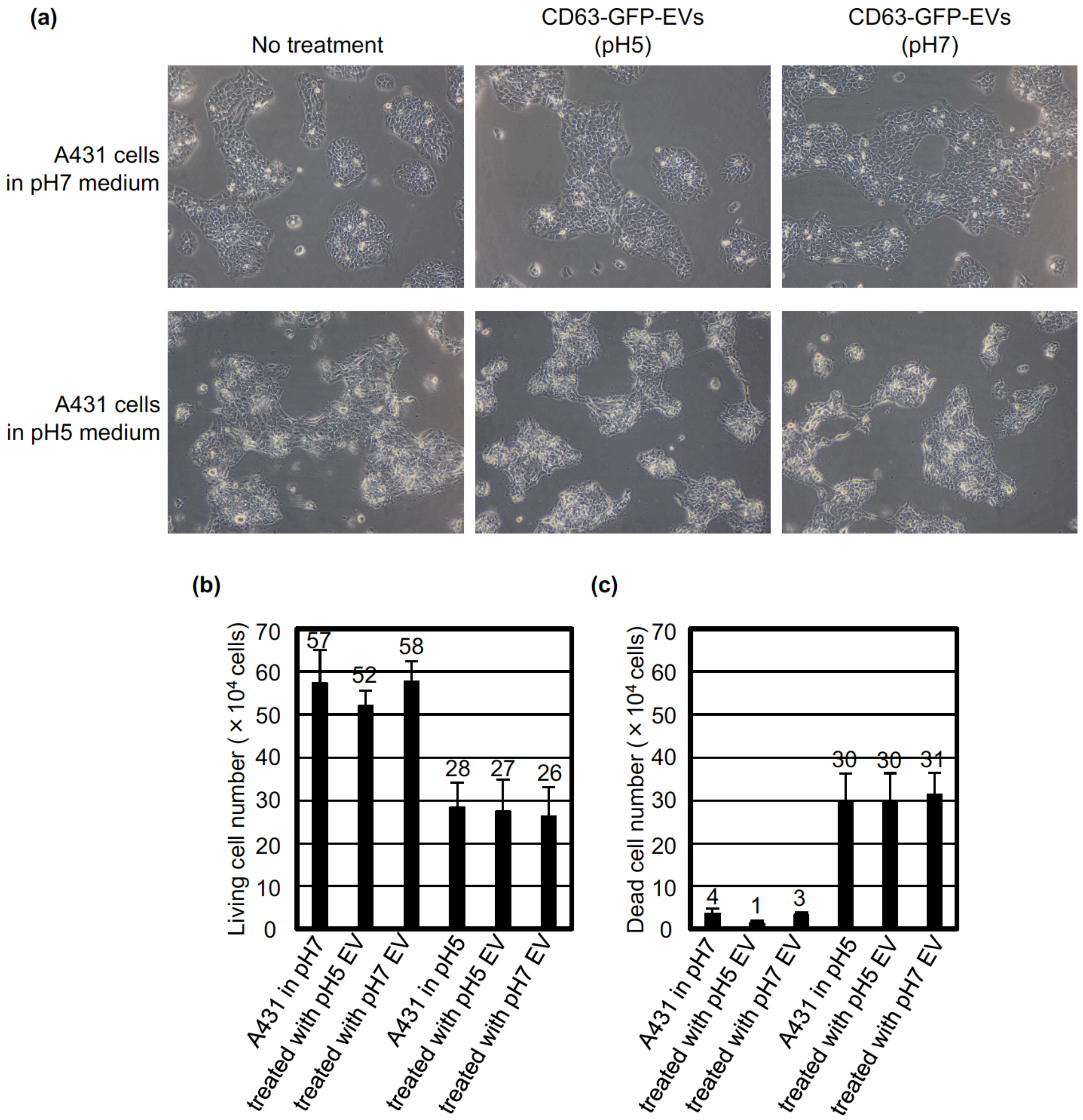

Supplementary Figure S5. Cell viability under cellular EV uptake experiments in serum-starved condition. (**a**) Microscopic observation of A431 cells treated with isolated CD63-GFP-EVs (20 μg/mL for 48 h at 37°C in MEM (pH 5 or 7) without FBS) secreted under pH 7 or pH 5 cell culture condition. (**b**, **c**) Living (**b**) or dead (**c**) cell counting assay after cellular EV uptake experiments of (**a**) in trypan blue staining method. The data are expressed as the mean (± SD) of three experiments.

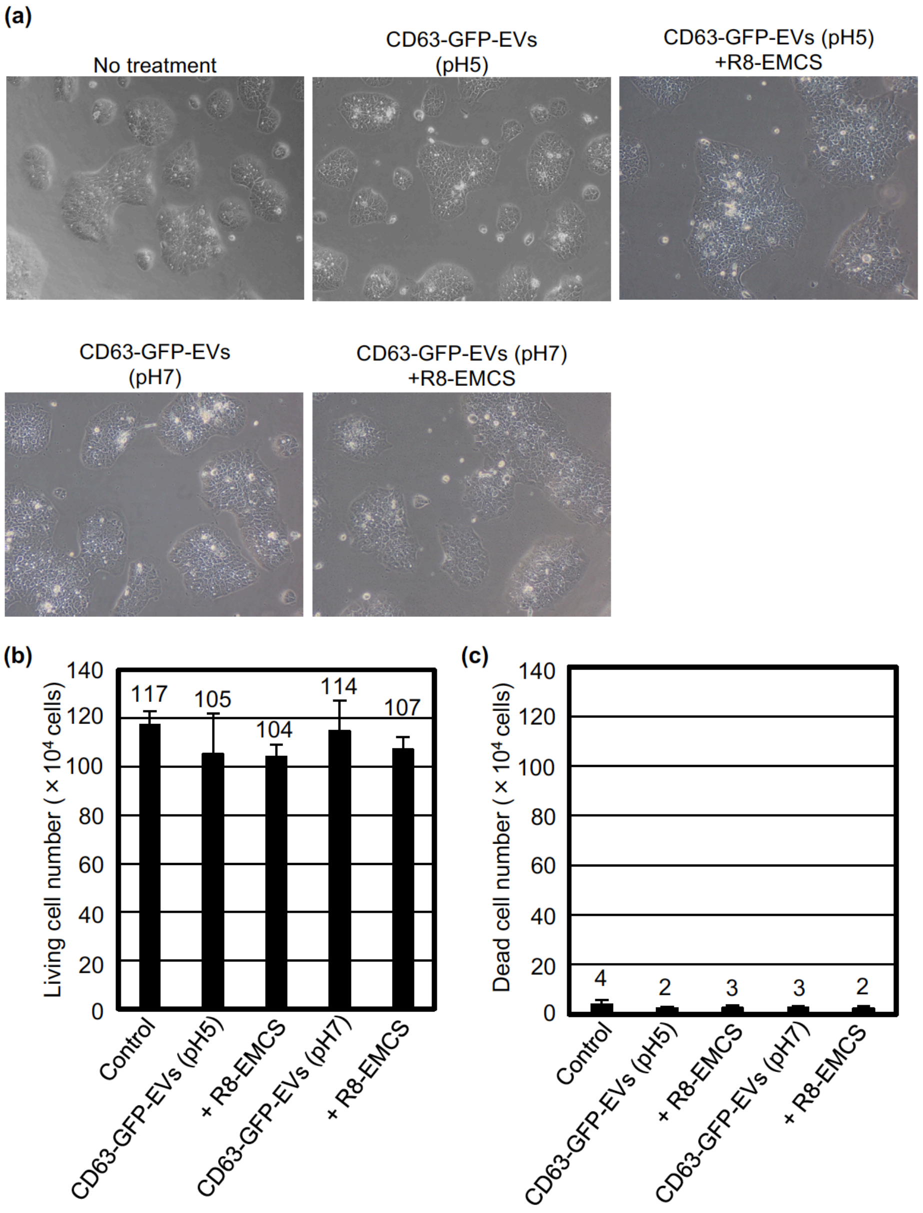

Supplementary Figure S6. Cell viability under cellular EV uptake experiments in 10% FBS containing condition. (**a**) Microscopic observation of A431 cells treated with isolated CD63-GFP-EVs (20 μg/mL for 24 h at 37°C in MEM (pH 7) with 10% FBS), secreted under pH 7 or pH 5 cell culture condition. (**b**, **c**) Living (**b**) or dead (**c**) cell counting assay after cellular EV uptake experiments of (**a**) by trypan blue staining. The data are expressed as the mean (± SD) of three experiments.
